# YdfD, a Lysis Protein of the Qin Prophage, Is a Specific Inhibitor of the IspG-Catalyzed Step in the MEP Pathway of *Escherichia coli*

**DOI:** 10.3390/ijms23031560

**Published:** 2022-01-29

**Authors:** Zhifang Lu, Biying Wang, Zhiyu Qiu, Ruiling Zhang, Jimin Zheng, Zongchao Jia

**Affiliations:** 1 College of Chemistry, Beijing Normal University, Beijing 100875, China; luzhifang@mail.bnu.edu.cn (Z.L.); 201831150034@mail.bnu.cn (B.W.); haoaqiu@163.com (Z.Q.); evelynsky@sina.cn (R.Z.); 2 Department of Biomedical and Molecular Sciences, Queen’s University, Kingston, ON K7L 3N6, Canada

**Keywords:** lysis, MEP pathway, prophage, IspG, YdfD

## Abstract

Bacterial cryptic prophage (defective prophage) genes are known to drastically influence host physiology, such as causing cell growth arrest or lysis, upon expression. Many phages encode lytic proteins to destroy the cell envelope. As natural antibiotics, only a few lysis target proteins were identified. *ydfD* is a lytic gene from the Qin cryptic prophage that encodes a 63-amino-acid protein, the ectopic expression of which in *Escherichia coli* can cause nearly complete cell lysis rapidly. The bacterial 2-*C*-methyl-D-erythritol 4-phosphate (MEP) pathway is responsible for synthesizing the isoprenoids uniquely required for sustaining bacterial growth. In this study, we provide evidence that YdfD can interact with IspG, a key enzyme involved in the MEP pathway, both in vivo and in vitro. We show that intact YdfD is required for the interaction with IspG to perform its lysis function and that the mRNA levels of *ydfD* increase significantly under certain stress conditions. Crucially, the cell lysis induced by YdfD can be abolished by the overexpression of *ispG* or the complementation of the IspG enzyme catalysis product methylerythritol 2,4-cyclodiphosphate. We propose that YdfD from the Qin cryptic prophage inhibits IspG to block the MEP pathway, leading to a compromised cell membrane and cell wall biosynthesis and eventual cell lysis.

## 1. Introduction

Cryptic prophages, also known as defective prophages, are segments of phage DNA integrated and maintained in bacterial chromosomes. It has been shown that bacterial genomes can harbor multiple cryptic prophages [1,2,3,4]. In fact, as much as 10–20% of bacterial chromosome segments are estimated to be prophage genes, and a growing body of evidence suggests that the proportion may be even greater [1,3]. For example, it was in the 1950s that the *E. coli* K-12 genomes were first found to carry phage genes [5] and now there are 10 known cryptic prophages residing in the chromosome of *E. coli* K-12 [4]. The expression of most of the prophage genes is usually suppressed under normal conditions, as certain proteins they encode are typically lytic and, therefore, toxic to the host [6]. However, there is evidence that some prophage genes can not only endow their hosts with traits and behaviors beneficial to surviving harsh environmental conditions but also expand the hosts’ genetic diversity, which plays a key role in microbial evolution [7,8,9,10]. Despite recent progress made in understanding cryptic prophages, the relationship between cryptic prophages and hosts is still largely elusive.

Investigation of cryptic prophages may provide hints to understand the process of phage infection, especially phage-induced cell lysis. As far as presently known, phages establish canonical lytic pathways to escape from hosts by two proteins, holin and endolysin [11,12,13]. Holins form holes in the cell membrane, and endolysins are a class of hydrolases that degrade bacterial cell wall. Therefore, holins are considered to control the access of endolysins to the cell periplasm. Apart from degradation of the cell wall, some phage genes encode proteins to inhibit the key enzyme to prevent cell wall biosynthesis. These proteins were dubbed “single-gene lysis” (Sgl) [11]. Many phages encode lytic protein. However, there are only three phage targets that have been proposed: (1) the Leviviridae Qβ uses the protein A2 to bind and inhibit MurA, which is the first step in bacterial cell wall biosynthesis [14]; (2) the protein E from phage ΦX174 inhibits peptidoglycan synthesis by interaction with MraY, which involves the peptidoglycan (PG) biosynthesis pathway [15]; and (3) LysM inhibits the translocation of the final lipid-linked PG precursor across the cytoplasmic membrane by interfering with MurJ [16]. Therefore, phages being natural antibiotics, exploring the lysis targets of phages is timely and of considerable importance.

Q-independent (Qin) prophage was the third prophage identified, found in *E. coli* K-12 [17]. As a part of Qin prophage, *ydfD* encodes a 63-amino-acid protein and *ydfD* is located downstream of *dicB*. Both *ydfD* and *dicB* are regulated by *dicA* [18]. Overexpression of *ydfD* induces cell lysis in *E. coli*, whereas lysis is prevented by coexpression with the cell division inhibitor *dicB* or *sulA*. It is now known that DicB as well as SulA prevent cell division by inhibiting FtsZ polymerization [19,20]. This indicates that the cell lysis caused by YdfD depends on functional cell division. Based on what is currently known, YdfD is speculated to promote cell lysis by targeting an unknown unique host protein, although the identity of the target protein of YdfD has not been established [21].

**Scheme 1:**
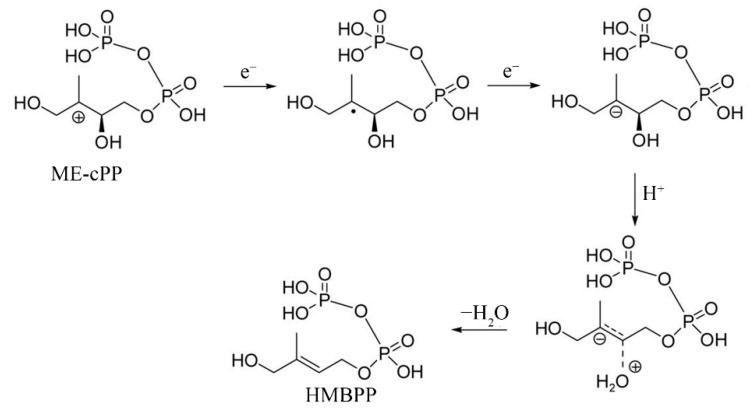
One of the proposed reaction mechanisms of IspG catalysis. (Adapted from Ref. [39]).

Our preliminary binding assays indicate that YdfD interacts directly with IspG, also known as GcpE [22]. IspG is a part of the 2-*C*-methyl-D-erythritol 4-phosphate (MEP) pathway, also called the 1-deoxy-D-xylulose 5-phosphate (DOXP) pathway, that occurs in eubacteria [23]. Most bacteria synthesize ubiquitous isoprenoid compounds via the MEP pathway. The MEP pathway products are isopentenyl pyrophosphate (IPP) and its isomer dimethylallyl pyrophosphate (DMAPP), both fundamental and unique five-carbon blocks for the synthesis of terpenoid compounds, such as dolichols and cholesterol, which are required for peptidoglycan synthesis and maintenance of cell membrane stability, respectively [24,25,26]. The MEP pathway proceeds through a linear sequence of seven enzymes. Understandably, the MEP pathway is completely absent in humans, and each of the seven enzymes has potential as a drug target [27,28,29,30,31,32]. Blocking the MEP pathway can be bactericidal, and multiple inhibitors have been developed [33,34,35]. Fosmidomycin, an inhibitor of 1-deoxy-D-xylulose 5-phosphate reductoisomerase (Dxr), is the most anticipated inhibitor because it is the sole MEP pathway inhibitor that is being investigated clinically [36,37,38]. The sixth, IspG, converts 4-diphosphocytidyl-methylerythritol 2-phosphate (ME-cPP) into methylerythritol 2,4-cyclodiphosphate (HMBPP) assisted by an Fe/S cluster [39] (Scheme 1). The conversion to HMBPP is an irreversible committed step in the MEP pathway. HMBPP is an essential precursor of cell wall and membrane synthesis, and, predictably, *E. coli* cells are unable to survive when the *ispG* gene is deleted [40,41,42].

Herein, we present the results of several in vitro experiments demonstrating the interaction between YdfD and IspG, including pull-down assays, size exclusion chromatography (SEC) analysis, and isothermal titration calorimetry (ITC). We confirm this interaction further in vivo using fluorescence resonance energy transfer (FRET) and by showing that the overexpression of *ispG* or the compensation of the IspG enzyme catalysis product HMBPP rescues cells from the rapid lysis induced by YdfD. Taking these together, we suggest that YdfD is a specific inhibitor of IspG and inhibits IspG-mediated synthesis of HMBPP. Thus, by targeting IspG, YdfD compromises cell wall biosynthesis and causes cell lysis.

## 2. Results

### 2.1. Protein-Protein Binding between YdfD and IspG

**Figure 1:**
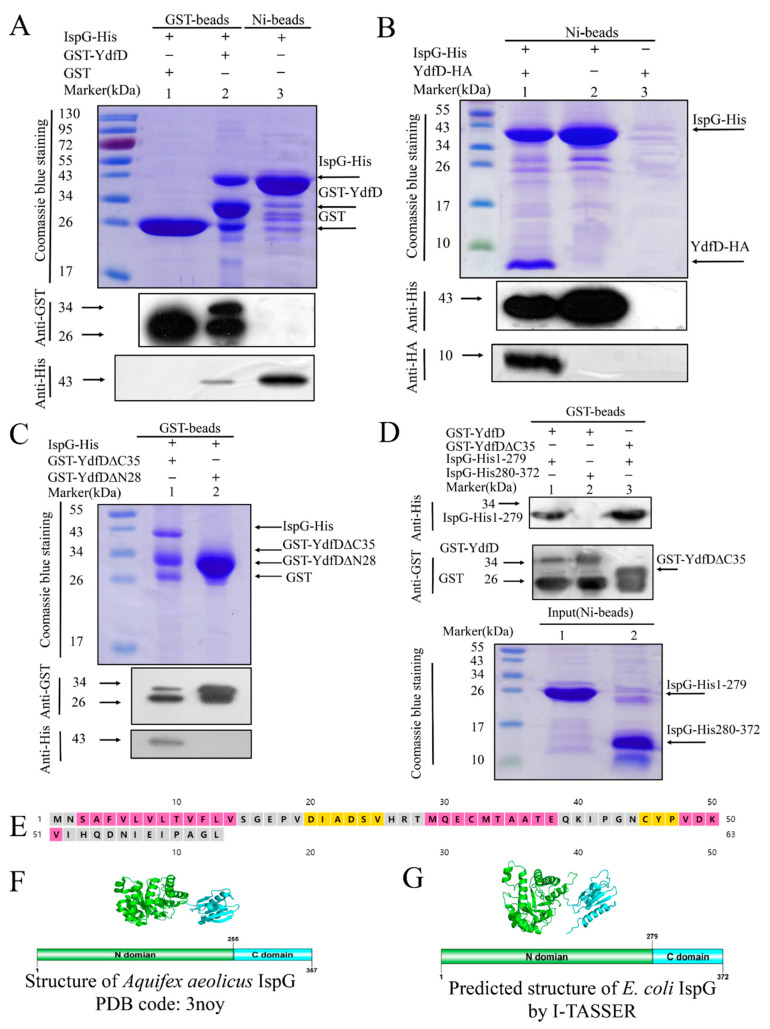
Pull-down assays between YdfD, IspG, and their derivatives. (**A**) Lane 1: GST control; lane 2: GST-YdfD pulled down IspG; lane 3: IspG was purified by Ni beads as a control; the arrows indicate the positions of the corresponding proteins. (**B**) Lane 1: IspG pulled down YdfD-HA; lane 2: IspG-His was purified by Ni beads as a control; lane 3: YdfD-HA was purified by Ni beads as a control; the arrows indicate the positions of the corresponding proteins. (**C**) Lane 1: GST-YdfD∆C35 pulled down IspG; lane 2: GST-YdfD∆N28 pulled down IspG. (**D**) Lane 1: GST-YdfD pulled down IspG 1-279; lane 2: GST-YdfD pulled down IspG 280-372; lane 3: GST-YdfD∆C35 pulled down IspG 1-279. (**E**) Predicted secondary structure of YdfD by the PSIPRED server. (**F**) The structure of *Aquifex aeolicus* IspG, PDB code: 3noy. (**G**) The predicted structure of *E. coli* IspG by I-TASSER.

Pull-down experiments were conducted to identify specific cellular targets of YdfD. His-YdfD was synthesized in *E. coli* BL21(DE3) cells as the bait for the whole cell lysate on Ni-NTA. After the Ni-NTA resin was washed, the eluted protein was subjected to SDS-PAGE and identified via mass spectrometry (MS). Dozens of proteins were identified, as shown in Table S1. Given YdfD’s function in inducing cell lysis, potential target proteins related to the cell membrane or cell wall synthesis were screened out. Next, we recombinantly expressed and purified these proteins and tested their interaction with YdfD with another pull-down assay (Table 1, Figure 1 and Figure S1).

Based on the MS and pulled-down results (Figure 1 and Figure S1), IspG stood out as the most likely to interact with YdfD. IspG, an oxidoreductase, is an essential enzyme in the MEP pathway that synthesizes vital precursors of the cell wall and membrane [41]. Given YdfD’s strong activity in inducing cell lysis [21], we selected IspG for further characterization. Further pull-down experiments using N-GST-tagged *ydfD* (Table S2) coexpressed with His-tagged *ispG* showed that both proteins were present in the elute following glutathione and Ni-NTA affinity chromatography, as confirmed by SDS-PAGE and Western blotting (Figure 1A,B). YdfD, therefore, appeared to exhibit strong binding to IspG, warranting further investigation of their interaction.

To identify the exact segments of YdfD and IspG involved in their interaction, multiple truncation derivatives of YdfD and IspG were generated (Table S2). YdfD is predicted by the PSIPRED server to consist of distinct two domains, C-terminal (residues 29–63) and N-terminal (residues 1–28) domains [43] (Figure 1E), so a C-terminal deletion mutant (YdfD∆C35) and an N-terminal deletion mutant (YdfD∆N28) were synthesized and purified. Pull-down results showed it was GST-YdfD∆C35 rather than GST-YdfD∆N28 that exhibited interaction with IspG (Figure 1C). 

Similarly, IspG constructs were designed using the structure of *Aquifex aeolicus* IspG (PDB code: 3noy) as a reference as it shares a 51.9% sequence identity with *E. coli* IspG. *Aquifex aeolicus* IspG has two separate domains, and as such, *E. coli* IspG is predicted using I-TASSER [44] to also consist of two distinct N-terminal (residues 1–279) and C-terminal (residues 280–372) domains (Figure 1F,G). As shown in Figure 1D, YdfD∆C35 showed interaction with IspG1-279. Although interactions of varying strength were detected for multiple derivatives, the interaction between the full-length YdfD and the full-length IspG was much stronger than that between the mutant combinations (Figure 1A,D), suggesting that competent IspG-YdfD interaction requires two full-length proteins.

### 2.2. YdfD Forms Stable Complex with IspG in Solution

**Figure 2:**
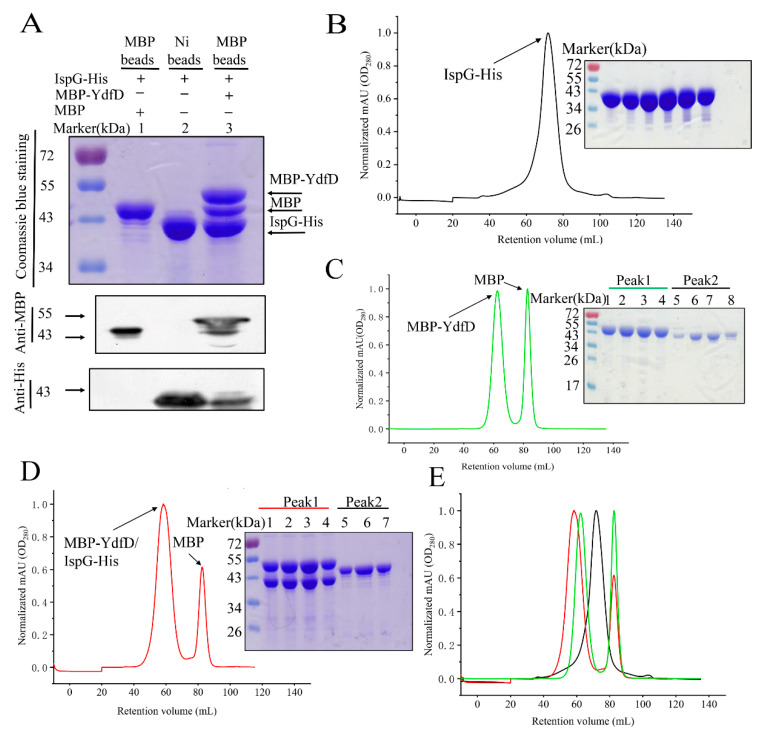
SEC (Superdex 200 10/300 GL; flow rate 1.0 mL/min; 280 nm) elution profiles of IspG, MBP-YdfD, and the IspG/MBP-YdfD complex. (**A**) Pull-down SDS and Western blotting; lane 1: MBP pulled down IspG and was purified by maltose protein binding resin as a control; lane 2: IspG protein was purified by Ni-NTA resin; lane 3: A complex of fusion protein MBP-YdfD and IspG was purified by the maltose protein binding resin; the arrows indicate the positions of the corresponding proteins. (**B**) Elution profile (left) and SDS-PAGE (right) of IspG. (**C**) Elution profile of MBP-YdfD (left) and SDS-PAGE (right) of the two peaks; lanes 1–4 correspond to the first peak (right), and lanes 5–8 correspond to the second peak (left). (**D**) Elution profile (left) of the MBP-YdfD/IspG complex and SDS-PAGE (right) of the two peaks; lanes 1–4 correspond to the first peak (right), and lanes 5–7 correspond to the second peak (left). (**E**) merged (**B**–**D**) elution profiles.

SEC was used to purify and analyze the YdfD/IspG complex. N-MBP-tagged YdfD and IspG-His were co-synthesized in *E. coli* B21(DE3) cells to purify a large amount of the YdfD and IspG complex (Figure 2A). MBP-YdfD, IspG-His, and their complex were individually loaded on a Superdex 200 SEC column. Individual IspG-His was observed at a retention volume of approximately 81 mL (Figure 2B). SDS-PAGE of the individual MBP-YdfD elution profile showed that the first peak was MBP-YdfD and the subsequent peak was MBP (Figure 2C). Compared with IspG-His and MBP-YdfD, the retention volume of the MBP-YdfD/IspG-His complex was 69 mL, earlier than either protein alone (Figure 2D,E), though the relative band intensities in SDS-PAGE gel for the two proteins co-eluted from the column were consistent. These results indicate a stable MBP-YdfD/IspG-His complex in solution.

### 2.3. YdfD and IspG Interactions In Vitro and Vivo

**Figure 3:**
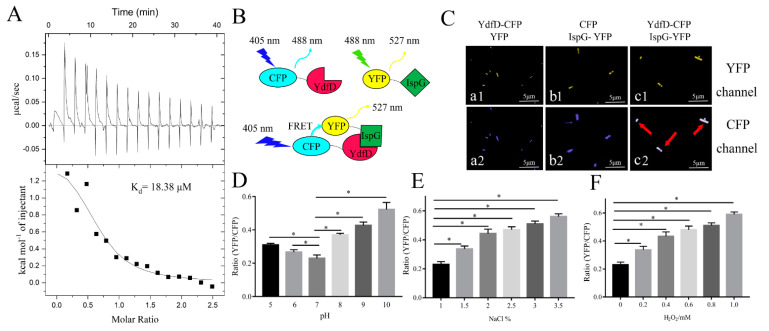
YdfD–IspG interactions in vitro and in vivo. (**A**) Binding curve of IspG titrated into YdfD via a single injection in identical buffer (20 mM Tris, 150 mM NaCl, pH = 8.0) detected by ITC; Kd of 18.38 μM was obtained by ITC. (**B**) A schematic representation of FRET. (**C**) Confocal imaging of FRET cells. (**a1**), YdfD-CFP and YFP were coexpressed and observed in the YFP channel; (**a2**), Ydfd-CFP and YFP were coexpressed and observed in the CFP channel; (**b1**), CFP and IspG-YFP were coexpressed and observed in the YFP channel; (**b2**), CFP and IspG-YFP were coexpressed and observed in the CFP channel; (**c1**), YdfD-CFP and IspG-YFP were coexpressed and observed in the YFP channel; (**c2**), YdfD-CFP and IspG-YFP were coexpressed and observed in the CFP channel; (**a1**,**a2**,**b1**,**b2**) were control. (**D**) Different pH values affected the emission intensity ratio (527/488 nm ratio) in living cells. (**E**) Different osmotic pressures affected the emission intensity ratio (527/488 nm ratio) in living cells. (**F**) Different oxidative levels affected the emission intensity ratio (527/488 nm ratio) in living cells. * refers to *p* < 0.05.

ITC is a powerful tool to measure enthalpy changes accompanying the binding of a compound or a protein to a protein [45]. ITC assays were used to quantify the affinity and thermodynamic parameters of the YdfD–IspG interaction. A considerable endothermic heat change was observed when YdfD was titrated with IspG via a single injection (Figure 3A). The best fit for the processed ITC data was obtained using a one-site binding equation [46]. The thermodynamic parameters between YdfD and IspG (Kd, n, ∆H, and ∆S) were 18.38 μM, 0.63, 1573 cal/mol, and 26.9 cal/mol/deg, respectively. The association constant (Kd, 18.38 μM) between YdfD and IspG is in micromolar range, indicating a relatively strong binding between YdfD and IspG.

Pull-down assays and ITC experiments demonstrated that YdfD associates with IspG in vitro. Further FRET experiments also demonstrated this interaction in vivo. *E. coli* BL21(DE3) cells were cotransformed with plasmids carrying *ydfD-cfp* and *ispG-yfp* and induced by IPTG for 8 h. Compared to the control (Figure 3C(a1,a2,b1,b2), the cells that were transformed with pET22b- *ydfD-cfp* and pET28b- *ispG-yfp* had distinct multiplex fluorescence signals under the CFP channel (Figure 3C(c2), indicated by the red arrow). The mixture fluorescence signals consisted of two components: the direct emission of CFP (Figure 3C(a2,b2)) and the emission of YFP excited by energy transferred from CFP, meaning that an energy transfer from CFP to YFP occurred upon YdfD interaction with IspG. In addition, FRET efficiency (ratio of YFP/CFP) increased when the cells were exposed to adverse conditions (Figure 3D–F).

### 2.4. The Intact YdfD Protein Is Required for Its Cell Lysis Function

**Figure 4:**
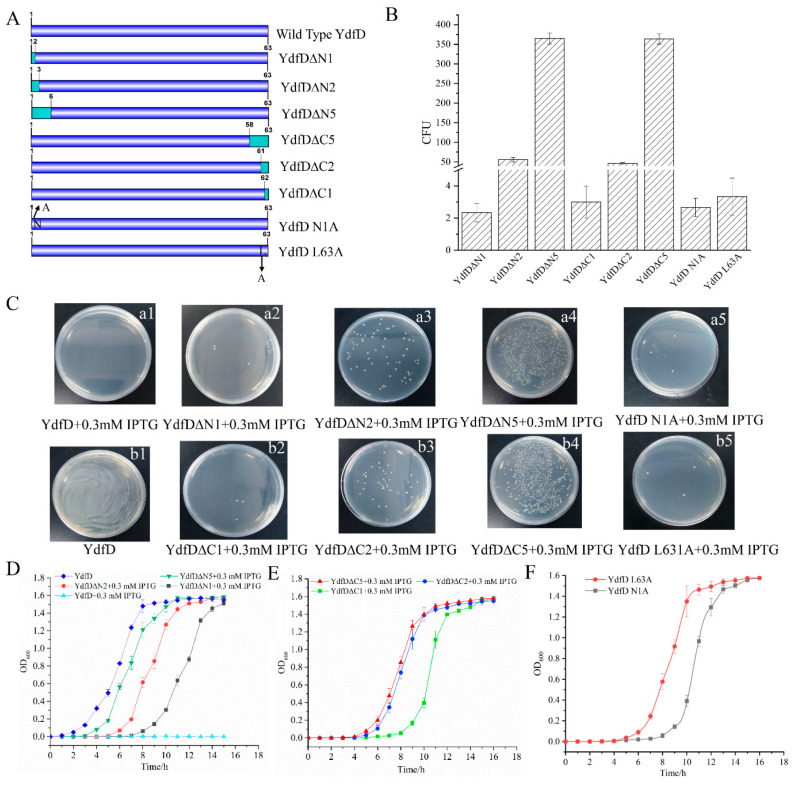
The YdfD N-terminus and C-terminus are critical to the lysis function. (**A**) Schematic of YdfD mutants. (**B**) CFU of YdfD mutants that survived on the plates. (**C**), (**a1**): Wild-type YdfD; cells could not survive on the plate; (**a2**): YdfD∆N1; (**a3**): YdfD∆N2; (**a4**): YdfD∆N5; (**a5**): YdfD N1A; (**b1**): YdfD (no IPTG induction); (**b2**): YdfD∆C1; (**b3**): YdfD∆C2; (**b4**): YdfD∆C5; (**b5**) YdfD L63A. (**D**) Growth curves of YdfD and mutants with induction by IPTG, wild-type in the absence of IPTG (blue), YdfD∆N5 (green), YdfD∆N2 (red), YdfD∆N1 (black), and wild-type YdfD (cyan). (**E**) Growth curves of YdfD mutants with induction by IPTG, YdfD∆C5 (red), YdfD∆C2 (blue), YdfD∆C1 (green), and wild-type YdfD (cyan). (**F**) Growth curves of YdfD mutants with induction by IPTG, YdfD N1A (red), and YdfD L63A (gray). All protein expression was induced by IPTG unless otherwise stated.

To investigate which segment of YdfD is responsible for its lysis activity, the N-terminal and C-terminal residue(s) was omitted stepwise and a series of YdfD mutants (YdfD∆C1, YdfD∆C2, YdfD∆C5, YdfD∆N1, YdfD∆N2, YdfD∆N5, YdfD N1A, and YdfD L63A) were produced (Figure 4A). Owing to the strong lysis activity of the IPTG-induced intact YdfD, cells overexpressing *ydfD* could not survive on LB plates (Figure 4C(a1)). However, all mutants survived on LB plates and longer deletions produced more CFUs (Figure 4B,C). In addition, compared with the intact YdfD, the strains with more residues omitted had lag phases shorter than those with fewer residues omitted and all strains reached similar OD_600_ maxima (Figure 4D–F). Although the N-terminal domain of YdfD interacted with IspG somewhat in vitro (Figure 1), it could not function individually in cells. Since the C-terminal domain of YdfD was also indispensable, it is plausible that the N-terminal domain and C-terminal domain of YdfD cooperate closely in its lysis function.

### 2.5. mRNA Levels of ydfD Increased under Stress

**Figure 5:**
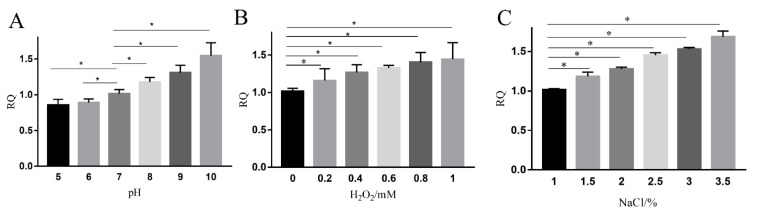
RT-PCR of *ydfD* in stress conditions. (**A**) pH gradient effect on the transcription levels of *ydfD*. (**B**) H_2_O_2_ gradient effect on the transcription levels of *ydfD*. (**C**) NaCl gradient effect on the transcription levels of *ydfD*; * *p* < 0.05.

Lysogenic bacteriophages enter the lytic cycle when the host is exposed to UV irradiation, X-ray irradiation, and mutagens [47,48,49], because the phage genes are activated, transcribed, and translated. To evaluate the effects of adverse conditions on the transcription of *ydfD* as part of prophage, we tested mRNA levels of *ydfD* in response to various stressors. RT-PCR showed that when *E. coli* cells were exposed to non-acidic stress conditions, including alkalinity (Figure 5A), oxidative stress (H_2_O_2_; Figure 5B), and high salinity (NaCl; Figure 5C), mRNA levels of *ydfD* increased compared to standard growth conditions (pH = 7, NaCl 1%, and absence of oxidative stress).

### 2.6. Compensation Experiments

**Figure 6:**
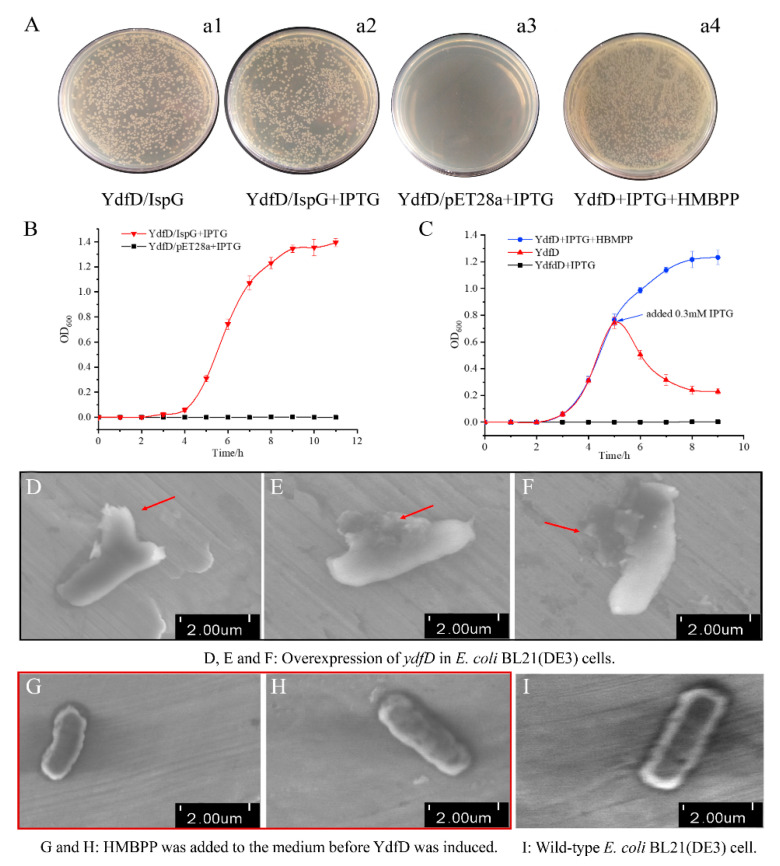
IspG or its catalysis product HMBPP were able to offset the lysis induced by YdfD. (**A**). (**a1**): *ydfD* and *ispG* were not induced; (**a2**): *ydfD* and *ispG* were induced; (**a3**): *ydfD* and an empty pET28a vector were co-induced; (**a4**): YdfD was synthesized in the presence of HMBPP. (**B**) Growth curves of the *ydfD*/*ispG* and *ydfD*/pET28a vector induced by IPTG. (**C**) Growth curves of *ydfD*-expressed cells with variations. (**D**–**F**) Overexpression of *ydfD* in *E. coli* BL21(DE3) cells, and as the red arrow shows, the cellular morphology became incomplete and abnormal. ((**G**,**H**): as the red box indicate) HMBPP was added to the medium before *ydfD* was induced, and HMBPP was able to rescue cell lysis. (**I**) Wild-type *E. coli* BL21(DE3) cell. Scale bar 2 μm.

After confirming the interaction between YdfD and IspG using multiple approaches, we attempted to determine if it was possible to offset YdfD-induced lysis either by coexpressing *ispG* with *ydfD* or by supplementing cells with the IspG enzyme catalysis product HMBPP (Figure 6A). Overexpressing *ispG* neutralized the lysis induced by overexpressed *ydfD* (Figure 6B), and supplementing with HMBPP completely restored normal cell growth (Figure 6C). The cell morphology was observed using SEM. The cell morphology was seriously compromised upon YdfD induction (Figure 6D–F) but remained unchanged in the presence of HMBPP (Figure 6G,H).

## 3. Discussion

The number of prophages identified in bacterial genomes or plasmids has increased substantially in recent years [4,45,46], although the underlying functional mechanisms of their host interactions have remained unclear [50]. Nevertheless, prophages appear to play key roles in host metabolism. Cryptic prophages have been shown to help bacteria resist antibiotic stress [50] and *dicB*, an upstream gene of *ydfD*, suppresses cell division in *E. coli* once induced [51]. Prophages can also prevent different categories of phages from infecting the host. DicB specifically inhibited infection by λ and other phages that use ManYZ membrane proteins to prevent phage DNA entrance [52], provide population-level benefits by promoting biofilm formation, and influence the evolution of their hosts [7,8,9].

A previous study showed that overexpression of *ydfD* caused cell lysis of *E. coli* [21], although the molecular mechanism was not illustrated. In this study, we attempted to characterize YdfD and its underlying mechanism of cell lysis activity. Herein, we report that YdfD interacts with the essential MEP enzyme IspG. The MEP pathway synthesizes precursors of the cell wall and membrane, and a variety of inhibitors have already been designed and screened against it [53,54]. It is thus tempting to suggest that YdfD-mediated cell lysis may be caused by YdfD inhibition of MEP production. This phenomenon may be regarded as a strategy for the prophage precursor to escape from the host under adverse conditions. This is analogous to the lytic function of protein E from phage ΦX174, which inhibits peptidoglycan synthesis by the MraY enzyme [15].

**Figure 7:**
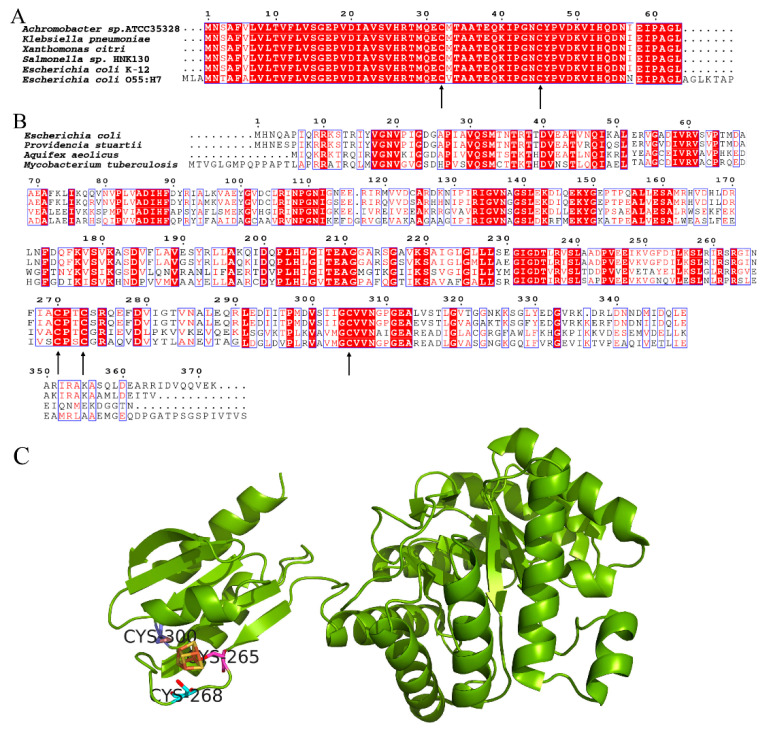
Conserved cysteine residues of YdfD and IspG. (**A**) Alignment of YdfD sequences from *Achromobacter* sp. ATCC35328, *Klebsiella pneumoniae*, *Xanthomonas citri*, *Salmonella* sp. HNK130, *E. coli* K-12, and *E. coli* O55:H7; the arrows indicate conserved cysteine residues. (**B**) Alignment of IspG sequences from *E. coli*, *Providencia stuartii*, *Aquifex aeolicus*, and *Mycobacterium tuberculosis*; the arrows indicate conserved cysteine residues. (**C**) *Aquifex aeolicus* IspG; three conserved cysteine residues binding the iron–sulfur cluster are responsible for the electron transfer of the catalytic process (PDB code: 3noy).

YdfD contains two well-conserved cysteine residues among bacterial species [21], and IspG has three conserved cysteine residues involved in the binding of the iron–sulfur cluster, responsible for the electron transfer of the catalytic process, as shown in Figure 7. The catalytic activity was significantly reduced when any cysteine that involved the cluster was replaced by serine [55]. However, the YdfD mutants lacking cysteine residue(s) had no effect on its binding to IspG (Figure S2), suggesting that the YdfD–IspG interaction is not through a disulfide bond. As the [4Fe-4S]^2+^ cluster is indispensable for IspG catalytic activity, all experimental procedures need to be performed under strict anerobic conditions [56]. In addition, ME-cPP, the substrate of IspG, is not commercially available. It is thus difficult to assay the effects of YdfD on IspG enzyme kinetics.

While it appears that the N-terminal domain of YdfD was responsible for binding to the N-terminal domain of IspG, intact YdfD was necessary for its full activity in the cell (Figure 4). It is possible that the N-terminal domain of YdfD recruits the C-terminal domain to IspG, which inhibits IspG’s activity (Figure 4), which is why both N-terminal and C-terminal domains of YdfD are essential for its cellular activity. This agrees with previous studies showing that the C-terminal domain of YdfD is essential for lysis but that the killing activity of the C-terminal domain alone is significantly reduced compared to that of the intact protein [21]. The *Aquifex aeolicus* IspG is known to fold into two domains: an N-terminal and a C-terminal domain [56]. In the *Aquifex aeolicus* IspG catalytic process, the substrate binds to the positively charged surface region at the N-terminal domain [57]. Our ProtParam model (https://web.expasy.org/protparam/, accessed on 10 November 2021) [58] shows that the C-terminal domain of YdfD is negatively charged. Hence, it is possible that the C-terminal domain of YdfD competitively inhibits IspG by obstructing the binding of its substrate, ME-cPP, to the positively charged surface region of the IspG N-terminal domain. As a result, cooperation of both YdfD domains would be essential to inhibit IspG activity.

Under normal conditions, lysogenic phage levels remain steady. However, once the environment worsens, the transcription of lysis genes increases as the lysogenic phages look for ways to escape from the host [59,60,61]. Prophages also respond in this manner: although they cannot successfully assemble into viable phage particles, the lysis genes (*ydfD*) of Qin would increase transcription under adverse conditions. As expected, the RT-PCR of *E. coli* grown under stress conditions showed that mRNA levels of *ydfD* dramatically increases in non-acidic stress conditions and only slightly decreases under acidic conditions (Figure 5). The slight transcription decrease in the acidic medium is likely due to the bacteria’s general preference for more alkaline conditions and the corresponding reduction in the growth rate in a medium with a pH of 5.0 [62,63]. Consequently, the transcription of all unessential genes would be expected to decrease when bacteria are grown in an acidic environment.

The products of the MEP pathway are IPP and DMAPP, both of which are sole precursors for vital cellular components, including hopanoids [64]; menaquinone [65]; and polyprenyl phosphates, particularly undecaprenyl diphosphate (Und-p), a lipid carrier to synthesize peptidoglycan cell wall precursors that are essential to maintain cell integrity [66,67,68]. No matter how complex the terpenoid compounds are, all of them share a common five-carbon isoprene building block. A likely mechanism for YdfD-induced lysis is that YdfD blocks the MEP pathway in the cell membrane and cell wall biosynthesis. This would result in a shortage of key precursors for the cell wall and the cell membrane, leading to the abortion of cell wall formation, especially during cell division. A previous study by Masudo et al. found that YdfD cannot induce cell lysis when cell division is restrained [21]. We suppose that YdfD cuts off the MEP pathway by interaction with IspG, resulting in deficient cell components and cell wall membrane. When *E. coli* cells divide normally, as cytomembrane and cell wall element synthesis is most intense at sites of division, YdfD blocks terpenoid synthesis, thus leading to cell lysis. However, when cell division is slowed or stalled, the need for terpenoids (e.g., Und-P) is reduced. Therefore, YdfD cannot lyse the nondividing cells, i.e., YdfD-mediated effects would depend on cell division [21]. Further studies are needed to explore the direct effects of YdfD on IspG. HMBPP synthesis is a committed step in the MEP pathway, and IspG is strongly conserved across bacterial species [39], so it is unsurprising that phages would adapt to target IspG specifically to disrupt the host physiology and exert their function. IspG will be the fourth identified target of the phage lysis proteins; the details of how YdfD binds IspG and interferes with HMBPP will likely require structure studies of the YdfD/IspG complex.

**Figure 8:**
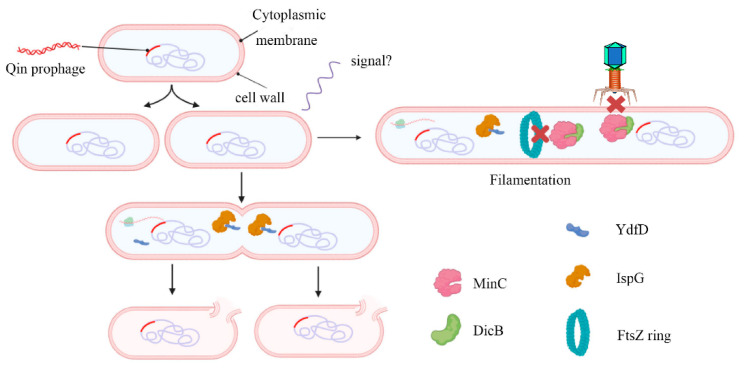
Working model for the regulation of host morphology by YdfD and DicB. The Qin cryptic prophage encodes the protein DicB and YdfD. When cell division is inhibited by DicB, demand for isoprenoid compounds is decreased and lysis is prevented (**right**). When YdfD is ectopically expressed, YdfD interacts with IspG, reducing isoprenoid production and causing the cells to lyse (**left**).

It is clear that DicB binds to MinC and prevents FtsZ ring formation. Moreover, DicB specifically inhibits infection by λ and other phages that use ManYZ membrane proteins to prevent phage DNA entrance [52]. *dicB* and *ydfD* are located in the same operon, with partial overlap. As mentioned before, DicB was able to offset YdfD-induced lysis [21]. We propose the following model: in normal conditions, DicB inhibits cell division and prevents phage infection; when the environment gets worse, the transcription and expression of *ydfD* are activated, eventually leading to cell lysis (Figure 8).

The growing severity of antibiotic resistance means there is a growing need to target novel bacterial pathways. The MEP pathway is absent in humans, making it an attractive target for antibiotic drug development [50]. The mechanisms used by prophages such as the Qin YdfD protein have proven to have a robust and widespread capacity to co-opt bacterial physiology. In addition to offering new insight into the particularities of host-prophage interactions, YdfD represents a promising new avenue for generating lead compounds for designing antibacterial agents.

## 4. Materials and Methods

### 4.1. Cell Strains and Plasmids Construction

*E. coli* DH5α and *E. coli* BL21(DE3) strains were purchased from Invitrogen. Plasmids were constructed and maintained in *E. coli* DH5α. All plasmids and primers used in this study are listed in Tables S2 and S3. *ydfD* and *ispG* were amplified by PCR from the *E. coli* BL21(DE3) cell genome. The DNA sequences of *ydfD* and *ispG* are shown in Table S3.

### 4.2. Protein Expression and Purification

All proteins were produced in *E. coli* BL21(DE3) cells. Competent cells were transformed by plasmids. The transformants were scraped from the plate and cultured in the Luria Bertani (LB) liquid medium (1% peptone, 0.5% yeast extract, 1% NaCl, pH = 7.0). Isopropyl-beta-D-thiogalactopyranoside (IPTG) was added to the final concentration of 0.3 mM when the optical density 600 nm (OD_600_) was about 0.8, and the strain was incubated for 20 h at 16 °C. Next, the cells were harvested and resuspended in lysis buffer (20 mM Tris, 150 mM NaCl, pH = 8.0) for high-pressure cell disruption. The debris was centrifugated at 15,000× *g* at 4 °C for 40 min, while the supernatants were incubated with a corresponding purification affinity beads resin. Maltose binding protein (MBP)-tagged, His-tagged, and glutathione S-transferase (GST)-tagged proteins were purified by amylose resin (NEB, Beverly, MA, USA), Ni-NTA (Qiagen, Duesseldorf, Germany) resin, and GST-tag (Beaver, Guangzhou, China) purification beads, respectively.

### 4.3. Mass Spectrometry

Mass spectrometry (MS) was used to identify the proteins bound to YdfD. *E. coli* BL21(DE3) cells synthesizing His-YdfD were subjected to induction with 0.3 mM IPTG at 16 °C for 4 h. The cells were harvested and resuspended in lysis buffer for cell disruption. The debris was centrifugated and the supernatants were subjected to incubation with Ni-NTA resin. Subsequently, the beads were washed 3 times with wash buffer (20 mM Tris, 250 mM NaCl, pH = 8.0) to remove non-specific binding proteins. The beads were analyzed by SDS-PAGE and digested with trypsin (Thermo Fisher Scientific, Waltham, MA, USA). The MS was performed as described [69]. After digestion, the tryptic peptides were desalted using C18 stage tips and separated by a C18 column (75 μm inner diameter, 150 mm length, 5 μm, 300 Å) with a Thermo-Dionex Ultimate 3000 HPLC system that was directly connected with a Thermo Scientific Q-Exactive HF-X Hybrid Quadrupole-Orbitrap mass spectrometer. The generated MS/MS spectra were searched against the Uniprot *E. coli* k-12(Taxon ID: 83333) database using the SEQUEST search engine with Proteome Discoverer 2.2 software. The search criteria were as follows: full tryptic specificity was required; one missed cleavage was allowed. Peptide spectral matches were validated using the percolator provided by Proteome Discoverer software based on the *q* values at a 1% false discovery rate.

### 4.4. Pull-Down Assays

Pull-down assays were performed to assess the interaction between YdfD and IspG. YdfD and its various mutants (Table S1) were attached to GST for the identification of YdfD–IspG interactions. For GST-YdfD pulled-down IspG-His, *E. coli* BL21(DE3) cells were cotransformed by pGEX-6P-1-*ydfD* and pET28a-*ispG-his* and induced by IPTG. After bacterial cells were harvested, they were lysed and centrifuged and the supernatants were incubated with the GST beads. Finally, the protein sample beads were directly subjected to SDS-PAGE and Western blotting (diluted 1:10) after 3 washes using wash buffer and heat denaturation (98 °C for 10 min). Other samples were prepared by the same method. To prepare large amounts of protein, MBP tag was fused to the N-terminus of YdfD. The complex of MBP-YdfD and IspG was purified via amylose resin; after the resin treatment with wash buffer, the protein was eluted by wash buffer containing 20 mM maltose. IspG protein was purified using His-tag by Ni-NTA resin and eluted by wash buffer containing 300 mM imidazole. Finally, the elution was concentrated by a centrifugal filter (Millipore, Billerica, MA, USA, 10 kD) to 1 mL and loaded onto a Superdex 200 column for SEC analysis. SEC runs were conducted at 4 °C at a flow rate of 1.0 mL/min using a buffer containing 20 mM Tris, 150 mM NaCl, and 0.5 mM DTT at pH = 8.0.

### 4.5. Cell Survival Assays

Cell survival assays were conducted to assess which structural segment(s) of YdfD is responsible for its lysis activity. *E. coli* BL21(DE3) cells were transformed by plasmids carrying *ydfD* or its mutants (Table S1). After being cultured overnight, the clones were transferred to the LB liquid medium and the culture was continued for 12 h. Next, the media were diluted to the same OD_600_. Colony forming unit (CFU) assays were performed by plating cells onto LB plates and counting the CFUs after culturing for 12 h. Similarly, for the growth assay, the strains were cultured in a fresh medium with the same inoculum. OD_600_ was measured every half an hour. The cell growth was evaluated by OD_600_ and CFUs. All cells were cultured at 37 °C unless otherwise specified. All assays were performed in triplicate.

### 4.6. Stress Experiments

Reverse transcriptase quantitative PCR assays were performed to measure the mRNA level of *ydfD* under different stress conditions. *E. coli* BL21(DE3) cells were cultured in an LB medium for 12 h, and subsequently the cultures were transferred to the LB medium (1:100) under different stress conditions and cultured for 12 h at 37 °C. The stress conditions tested included pH values of the medium changing from 5.0 to 10.0, with a gradient of 1.0; increased H_2_O_2_ concentration of the medium from 0 to 1 mM, with a gradient of 0.2 mM; and increased NaCl concentration of the medium from 1 to 3.5%, with a gradient of 0.5% [47,60,67]. mRNA isolation was performed according to kit protocols (Tiangen Biotech, Beijing, China). Next, the DNAase was added to the mRNA to digest the DNA. Finally, the mRNA was reverse-transcripted at 42 °C for 30 min and real-time PCR assays were performed as described [70]. The primer sequences are listed in Table S3, and the 16S rRNA gene was used as a control. All assays were performed in triplicate.

### 4.7. Fluorescence Resonance Energy Transfer

FRET assays were performed to measure the interaction between YdfD and IspG in cells. The donor cyan fluorescent protein (CFP) was fused to the C-terminus of YdfD, and the receptor yellow fluorescent protein (YFP) was attached to the C-terminus of IspG (Table S2). *E. coli* BL21(DE3) cells were cotransformed by the plasmids of pET22b-*ydfD-cfp* and pET28a-*ispG-yfp*, and the fusion proteins were induced by IPTG for 8 h at 37 °C. The cells were dropped onto microslides for observation. For CFP, the excitation and emission wavelengths were 405 and 488 nm, respectively; for YFP, the excitation and emission wavelengths were 488 and 527 nm, respectively; and for FRET, the excitation and emission wavelengths were 488 and 527 nm, respectively (Zeiss LSM980 Airyscan 2, Oberkochen, Germany). Additionally, we quantified the YFP/CFP ratio by detecting the fluorescence intensity at 488 and 527 nm for 30 s.

### 4.8. Isothermal Titration Calorimetry

ITC (GE, Microcal iTC-200, Boston, MA, USA) assays were used to further characterize and quantify the interaction between YdfD and IspG. MBP-YdfD and IspG protein were prepared as mentioned above. For YdfD, the MBP tag was cleaved by PreScission proteases. YdfD and IspG were purified and concentrated in buffer (20 mM Tris, 150 mM NaCl, pH = 8.0) to 90 μM and 1.1 mM, respectively. Single-injection measurements were collected by injecting 2 μL of 1.1 mM IspG into 0.9 μM YdfD at a spacing of 120 s. All assays were run at 25 °C at a stirring speed of 300 rpm. The raw data were processed by Origin Pro 2018 to obtain the binding parameters between YdfD and IspG, including the association constant (K_d_), the enthalpy value (∆H), and the entropy value (∆S). All assays were performed in triplicate.

### 4.9. Compensation Experiment

Rescue experiments contained two independent experiments. The first experiment was performed to test whether the overexpression of *ispG* in vivo could prevent the cell lysis induced by YdfD. To achieve this goal, *ydfD* and *ispG* were overexpressed into *E. coli* BL21(DE3) cells and their expression were induced simultaneously by IPTG as above. In the second experiment, to explore whether the IspG catalysis product was able to prevent cell lysis, the IspG enzyme catalysis product HMBPP (Sigma, St. Louis, MO, USA) was added to the medium. Cell growth was evaluated by monitoring OD_600_ and CFUs in plates. For the test, 0.2 mg of HMBPP was added to 10 mL of the LB culture medium. Since HMBPP is largely insoluble in the LB medium, we did not note its accurate concentration.

### 4.10. Scanning Electron Microscopy

SEM analysis was used to observe the bacterial morphology [71]. The plasmids were transformed into *E. coli* BL21(DE3) cells and induced by 0.3 mM IPTG for 8 h. The cells were collected by centrifugation at 3000× *g* for 10 min. After the cells were washed with PBS (pH = 7.4), they were resuspended using 2.5% glutaraldehyde in 0.1 M cacodylate buffer and gently shaken at 37 °C for 30 min. The cells were dropped onto a foil after dehydration by gradient acetone solutions (10%, 30%, 50%, and 70%, 20 min each). Eventually, the foil was fastened onto a sample holder by conductive tape and the pictures of bacteria were captured by SEM (Hitachi SU8010, Toyko, Japan) at 5 kV and 3 μA.

## Figures and Tables

**Table 1:** Pull-down confirmation of interaction with YdfD of target proteins identified via MS.

Accession	Protein	Physiological Function or Processes	Interaction with YdfD
A0A029II96	GlmS	Hexosamine biosynthesis	No
E1JEU9	ErpA	A-type iron–sulfur carrier	No
A7ZNX6	FolE	Tetrahydrofolate biosynthesis	No
A0A070S6E1	IspG	MEP pathway	Yes
A0A017JTH0	IscS	Cysteine desulfurase	No
D6I572	LpxA	Lipid biosynthesis	No
M8THF9	CydA	Ubiquinol oxidase	No
A0A017IFA8	KdsA	Lipopolysaccharide biosynthesis	No

## Data Availability

The datasets of this study are available from the corresponding author on reasonable request.

## Supplementary Materials

The following supporting information can be downloaded at: https://www.mdpi.com/article/10.3390/ijms23031560/s1.
