# Supplementary material for: YdfD, a Lysis Protein of the Qin Prophage, Is a Specific Inhibitor of the IspG-Catalyzed Step in the MEP Pathway of Escherichia coli
Source: Int J Mol Sci. 2022 Jan 29;23(3):1560. doi: 10.3390/ijms23031560 (PMC8835842; doi:10.3390/ijms23031560)
Supplement: Supplementary file 1 [file ijms-23-01560-s001.zip › ijms-1539307 Supplementary Materials_edited.pdf]

Supplementary Materials

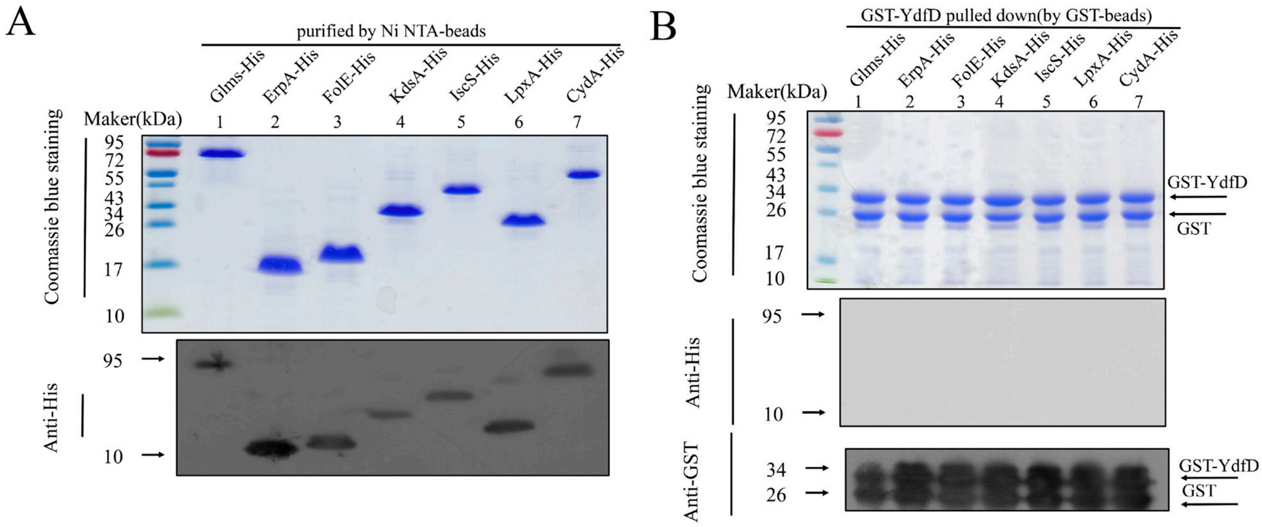

Figure S1. Pull-down assays between YdfD and potential target proteins. (**A**), lane 1: GlmS-His; lane 2: ErpA-His; lane 3: FolE-His; lane 4: KdsA-His; lane 5: IscS-His; lane 6: LpxA-His; lane 7: CydA-His, all the seven proteins were purified by Ni beads; (**B**), lane 1: GlmS-His; lane 2: ErpA-His; lane 3: FolE-His; lane 4: KdsA-His; lane 5: IscS-His; lane 6: LpxA-His; lane 7: CydA-His, all the seven proteins were pulled down by GST-YdfD. The results showed that GST-YdfD did not interact with above-mentioned proteins.

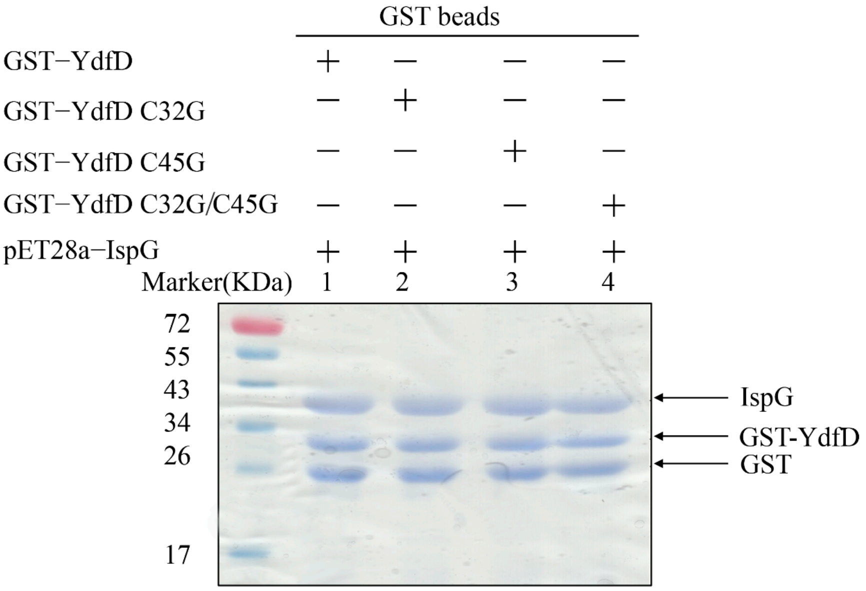

Figure S2. Pull-down assays between YdfD or its derivatives with IspG. Lane1: Wild type YdfD pulled down IspG, lan 2: YdfD C32G pulled down IspG, lane 3: YdfD C45G pulled down IspG, lane 4: YdfD C32G/C45G pulled down IspG. The results showed that a disulfide bond did not form between the YdfD and IspG.

Table S2. Plasmids used in this study.

Plasmid	Genotype	Restriction site
pET22b	P_{T7}, Amp^r, *ori*$_{pBR322}$, *lac*I	
pET22b-*ydfD*	*ydfD* cloned on pET22b	Nde I /Xho I
pET22b-*ydfD-HA*	*ydfD* cloned on pET22b with HA tag	Nde I /Xho I
pET22b-*his-ydfD*	*ydfD* cloned on pET22b with His tag	Nde I /Xho I
pET22b-*ydfD* C1	*ydfD* C1 cloned on pET22b	Nde I /Xho I
pET22b- *ydfD* C2	*ydfD* C2 cloned on pET22b	Nde I /Xho I
pET22b- *ydfD* C5	*ydfD* C5 cloned on pET22b	Nde I /Xho I
pET22b- *ydfD* N1	*ydfD* N1 cloned on pET22b	Nde I /Xho I
pET22b- *ydfD* N2	*ydfD* N2 cloned on pET22b	Nde I /Xho I
pET22b- *ydfD* N5	*ydfD* N5 cloned on pET22b	Nde I /Xho I
pGEX-6P-1	Ptac, Amp^r, *ori*$_{pBR322}$, *lac*I	
pGEX-6P-1- *ydfD*	*ydfD* cloned on pGEX-6p-1	BamH I /Xho I
pGEX-6P-1- *ydfD* N28	*ydfD* N28 cloned on pGEX-6p-1	BamH I /Xho I
pGEX-6P-1- *ydfD* C35	*ydfD* C35 cloned on pGEX-6p-1	BamH I /Xho I
pGEX-6P-1- *ydfD* C32G	*YdfD* C32G cloned on pGEX-6p-1	BamH I /Xho I
pGEX-6P-1- *ydfD* C45G	*YdfD* C45G cloned on pGEX-6p-1	BamH I /Xho I
pGEX-6P-1- *ydfD* C32G/C45G	*YdfD* C32G/C45g cloned on pGEX-6p-1	BamH I /Xho I
pET22b-*mbp-ydfD*	*mbp-YdfD* cloned on pET22b	Nde I /Xho I
pET28a	P_{T7}, Kan^r, *ori*$_{pBR322}$, *lac*I	
pET28a-*ispG-his*	*ispG* cloned on pET28a with His tag	Nco I /Xho I
pET28a-*ispG-his*1-279	*ispG* 1-279 cloned on pET28a with His tag	Nco I /Xho I
pET28a-*ispG-his* 280-372	*ispG*280-372 cloned on pET28a with His tag	Nco I /Xho I
pET22b-*ydfD-cfp*	*ydfD-cfp* cloned on pET22b	Nde I /Xho I
pET28a-*ispG-yfp*	*ispG-yfp* cloned on pET28a	Nco I /Xho I

Table S3. Primer and gene sequence

Primer and gene sequence	Sequence (5′-3′)
pET22b-*ydfD* 5F	CATATGAATTCAGCATTTGTGCTT
pET22b-*ydfD* 3R	CTCGAGAAGACCTGCCGGGATT
pET22b-y*dfDHA* 3R	CTCGAGAGCGTAGTCTGGGACGTCGTATGGGTAAAGACCTGCCGGGATTT
pET22b-*ydfD* C13R	CTCGAGACCTGCCGGGATTTCGA
pET22b-*ydfD* C2 3R	CTCGAGTGCCGGGATTTCGATATT
pET22b-*ydfD* C5 3R	CTCGAGTTCGATATTATCCTGGTG
pET22b- *ydfD* N1 5F	CATATGTCAGCATTTGTGCTT
pET22b- *ydfD* N2 5F	CATATGGCATTTGTGCTTGTT
pET22b- *ydfD* N5 5F	CATATGCTTGTTCTGACAGTT
pGEX-6P-1- *ydfD* 5F	GGATCCATGAATTCAGCATTTG
pGEX-6P-1- *ydfD* 3R	CTCGAGTTCGATATTATCCTGGT
pGEX-6P-1- *ydfD* N28 5F	CTCGAGACAATGCAGGAGTGTATG
pGEX-6P-1- *ydfD* C35 3R	CTCGAGTCATTACATTGTCCTGTGAACA
pGEX-6P-1- *ydfD* C32G 5F	CAGTGTTCACAGGACAATGCAGGAGGGTATGACTGCAGCAA
pGEX-6P-1- *ydfD* C32G 3R	TTCTGTTCGGTTGCTGCAGTCATACCCTCCTGCATTGTCCT
pGEX-6P-1- *ydfD* C45G 5F	AACCGAACAGAAAATTCCCGGTAACGGTTACCCGGTCGATA
pGEX-6P-1- *ydfD* C45G 3R	GGTGAATAACTTTATCGACCGGGTAACCGTTACCGGGAATTT
pET28a-*ispG*-5F	CCATGGCGATGCATAACCAGGCTCCA
pET28a-*ispG*-3R	CTCGAGTTTTTCAACCTGCTGAAC
pET28a-*ispG-his*1-279 3R	CTCGAGATCAAATTCCTGACGCGAA
pET28a-*ispG-his* 280-372 5F	CCATGGCGATGGTTATCGGTACGG
pET22b-*ydfD-cfp* 3R	CTCGAGCTTGTACAGCTCGTCCAT
RTPCR-*ydfD*-F	AGCATTTGTGCTTGTTCT

RTPCR-*ydfD*-R	TGCTGCAGTCATACACTC
RTPCR-16S-F	TGCTGCAGTCATACACTC
RTPCR-16S-R	GGCAGTTTCCCAGACATTAC
ydfD	ATGAATTCAGCATTTGTGCTTGTTCTGACAGTTTTTCTTGTTTCCGGA-<br>GAGCCAGTTGATATTGCAGACAGTGTTCACAGGACAATGCAGGAGTG-<br>TATGACTGCAGCAACCGAACAGAAAATTCCCGGTAACTGTTACCCGGTCGA-<br>TAAAGTTATTCACCAGGATAATATCGAAATCCCGGCAGGTCTT
ispG	ATGCATAACCAGGCTCCAATTCAACGTAGAAAATCAACACGTATTTACGTT-<br>GGGAATGTGCCGATTGGCGATGGTGCTCCCATCGCCGTACAGTCCATGAC-<br>CAATACGCGTACGACAGACGTCGAAGCAAC-<br>GGTCAATCAAATCAAGGCGCTGGAACGCGTTGGCGCTGATATCGTCCGTG-<br>TATCCGTACCGACGATGGACGCGGCAGAAGCGTTCAAACTCATCAAACAG-<br>CAGGTTAACGTGCCGCTGGTGGCTGACATCCACTTCGACTATCGCATT-<br>GCGCTGAAAGTAGCGGAATACGGCGTCGATTGTCTGCGTATTAACCCTGG-<br>CAATATCGGTAATGAAGAGCGTATTCGCATGGTGGTTGACTGTGCGCGCGA-<br>TAAAAACATTCCGATCCGTATTGGCGTTAACGCCGGATCGCTGGAAAAA-<br>GATCTGCAAGAAAAGTATGGCGAACCGACGCCGCAGGCGTTGCTG-<br>GAATCTGCCATGCGTCATGTTGATCATCTCGATCGCCTGAACTTCGATCAG-<br>TTCAAAGTCAGCGTGAAAGCGTCTGACGTCTTCCTCGCTGTTGAG-<br>TCTTATCGTTTGCTGGCAAAACAGATCGATCAGCCGTTGCATCTGGGGATCAC-<br>CGAAGCCGGTGGTGCGCGCAGCGGGGCAGTAAAATCCGCCATT-<br>GGTTTAGGTCTGCTGCTGTCTGAAGGCATCGGCGACACGCTGCGCG-<br>TATCGCTGGCGGCCGATCCGGTCGAAGAGATCAAAGTCGGTTTCGATATTTT-<br>GAAATCGCTGCGTATCCGTTCGCGAGGGATCAACTTCATCGCCTGCCCGAC-<br>CTGTTCGCGTCAGGAATTTGATGTTATCGGTACGGTTAACGCGCTGGAGCAAC-<br>GCCTGGAAGATATCATCACTCCGATGGACGTTTCGAT-<br>TATCGGCTGCGTGGTGAATGGCCCAGGTGAGGCGCTGGTTTCTACAC-<br>TCGGCGTCACCGGCGGCAACAAGAAAAGCGGCCTCTATGAA-<br>GATGGCGTGCGCAAAGACCGTCTGGACAACAACGATATGATCGACCAGCTG-<br>GAAGCACGCATTCGTGCGAAAGCCAGTCAGCTGGACGAAGCGCGTCGAATT-<br>GACGTTCAGCAGGTTGAAAAA
